# A Culturally Tailored Digital Education Intervention to Improve Nutrition Among Older Adult Congregate Meal Participants During COVID-19: Protocol for a Stepped-Wedge Cluster Randomized Controlled Trial

**DOI:** 10.2196/65976

**Published:** 2025-09-25

**Authors:** Vidya Sharma, Michelle Aguilar, Salma Abdelrahman, Erica Sosa, Meizi He, M Marilu Martinez, Andrea Hutson, Tianou Zhang, Zenong Yin, Sarah Ullevig

**Affiliations:** 1 Nutrition and Dietetics Program, College for Health, Community and Policy, University of Texas at San Antonio, 1 UTSA Circle, San Antonio, TX, 78249, United States, 1 2104587034; 2 Department of Public Health, College for Health, Community and Policy, University of Texas at San Antonio, San Antonio, TX, United States; 3 The Institute for Health, Community and Policy, University of Texas at San Antonio, San Antonio, TX, United States; 4 Agile Analytics, LLC, Austin, TX, United States; 5 Department of Health and Exercise Sciences, University of the Pacific, Stockton, CA, United States

**Keywords:** digital divide, older adults, elderly, nutrition, technology, digital education, United States, COVID-19

## Abstract

**Background:**

Inadequate nutrition and a lack of physical activity contribute to functional decline and complications from chronic diseases in older adults. The pandemic halted or altered necessary Older Americans Act (OAA) nutrition services provided to vulnerable, community-dwelling older adults in San Antonio, Texas. The “digital divide” or gap in technological access and knowledge further heightened the detrimental effect of the COVID-19 pandemic on older adults who may be “digitally excluded” from social, economic, and health-related interactions. During the pandemic, San Antonio congregate meal sites funded by OAA remained partially open biweekly to distribute meals but no longer offered in-person nutrition education, physical activity classes, and social activities. This project expands the current congregate meal programming infrastructure and partnerships with Older Adults Technology Services (OATS) to create a sustainable approach focused on improving the health of older adults.

**Objective:**

The study aims (1) to test the impact of a digital nutrition education intervention on the primary outcomes of food security and diet quality; (2) to determine the effect of the intervention on secondary outcomes of technology knowledge and usage, physical activity, and social isolation and loneliness; and (3) to examine the long-term impact and sustainability of technology use on food security, diet quality, physical activity, social isolation, and loneliness.

**Methods:**

This proposed digital nutrition education intervention study targets technologically limited older adults enrolled in the congregate meal program (CMP) using a stepped-wedge clustered randomized controlled trial. Key community partners, City of San Antonio Department of Health Services Senior Services Division and OATS, contributed to the study’s planning phase, research design, and implementation. The 20-week intervention included 5 weeks of in-person technology training, including internet access and technical support for 1 year and devices, followed by 15 weeks of a culturally tailored online nutrition education intervention. The study randomized 398 older adults from 12 congregate meal sites. Data collection took place at baseline, 3 months, 6 months, 9 months, 12 months, and 18 months. If successful, the impact of this program could be applied throughout the national OATS network and to similar CMPs to bridge the digital divide beyond the COVID-19 pandemic.

**Results:**

Recruitment and enrollment of 398 older adults at 12 CMPs was completed in December 2022. Study CMPs were randomly assigned to Cohort 1 and 2: 164 completed Cohort 1 in August 2023 and 111 completed Cohort 2 in April 2024. Eighteen-month data collection is ongoing.

**Conclusions:**

This study aims to determine the impact of a digital nutrition intervention on older adults’ nutrition status, physical activity, loneliness and isolation, and technology access and usage. Results from this study can inform future interventions with vulnerable populations and may serve as a basis for other OAA nutrition services.

## Introduction

According to the 2020 population estimates from the Census Bureau, the fastest-growing demographic consists of adults aged 65 years or older, totaling over 55 million nationwide. Texas has a large number of older adult residents (3.9 million), second only to California and Florida [1]. Nutrition is essential for healthy aging [2], the maintenance of health, and the prevention of chronic diseases. However, older adults are at an increased nutritional risk due to a unique set of circumstances that encompasses physiological, cognitive, and social factors, including loneliness, depression, lack of money for food and access to food, and physical impairments that hinder food procurement and preparation [3]. Factors such as food insecurity and malnutrition contribute to suboptimal nutrition and elevate the risk of chronic diseases [4]. Research indicates that food insecurity rates among older adults can be as high as 20% [5], with malnutrition rates reaching 27% among community-dwelling older adults [5,6]. Poor diet quality has been correlated with diminished functional capabilities, heightened susceptibility to falls [7], frailty [8], and increased mortality [9]. Therefore, addressing food security among older adults at risk is imperative for enhancing dietary standards, preventing malnutrition and its associated ramifications, and managing chronic conditions.

The goals of the congregate meal program (CMP) funded by the Older Americans Act (OAA) are to reduce hunger and food insecurity, promote health and socialization, and delay adverse health effects so community-dwelling older adults 60 years or older may remain independent in their homes, reducing institutionalization [10]. Socialization at congregate meal centers provides mental well-being benefits in addition to nutritional benefits. The CMP serves lunchtime meals for up to 5 days per week to over 1.5 million older adults in the United States and served over 60,000 older adults in Texas in 2018 [11]. Although the CMP meals provide one-third of the dietary reference intakes, allow for higher intakes of certain nutrients, and contribute to overall higher diet quality compared to nonparticipants, nutrient inadequacies still exist, especially among lower-income participants [12]. Gaps in technological access and knowledge referred to as the “digital divide” [13] disproportionally affect older adults, those who live in rural areas, the disabled, and the impoverished populations [14]. The COVID-19 pandemic heightened the awareness of the digital divide and deepened inequities in vulnerable populations [15]. In addition, the COVID-19 pandemic amplified existing challenges for older adults in maintaining their health. Marginalized and vulnerable older adults experiencing poverty, chronic conditions, financial instability, and limited technology access and knowledge before the pandemic shouldered much of the pandemic burden and risk [16-19]. Importantly, at-risk community-dwelling older adults who attend congregate meal sites lack digital nutrition interventions to address deficits in suspended services [20]. Eating alone is a nutritional risk factor and is associated with reduced food intake [21] and risk for malnutrition. With centers closed, those individuals who live alone are impacted by social isolation to a greater extent, and providing an online social environment during meal times can alleviate this risk factor and could potentially improve nutrient status. Thus, improving technology access and knowledge and delivering a virtual nutrition intervention can extend beyond the constraints of CMP participants to other vulnerable older adult populations. Although online services may never fully replace in-person interactions, the overall reliance and dependency on technology have become an integral part of our society.

Successful dietary behavioral change interventions with older adults have common features including using behavior change theories to promote self-efficacy, containing clear and repeated messages, incorporating incentives for motivation, and sufficient contact [22,23]. Positive behavior change has successfully increased fruit and vegetable intake and fiber in interventions targeting community-dwelling older adults [24]. Interventions at congregate meal sites have successfully increased participant fruit and vegetable intake after a 5-session program [25] or a 4-week intervention [26]. Additional studies have documented improvements in nutrition risk scores, a small improvement in food-related behaviors for both congregate and homebound meal participants [27], and improvements in dietary supplement knowledge and behaviors in congregate meal participants [28].

Limited studies on digital interventions for older adults impacting nutrition exist, and published studies focus on eHealth or Telehealth [29,30] and disease-specific interventions [31-35]. Successful digital interventions have been documented using “telemonitoring” for 4 months to improve nutritional status and increase vegetable, fruit, and dietary fiber intake in a Dutch population [30]. This study observed a high level of participant satisfaction, with an 80% completion rate. The majority of dropouts were older and had poorer cognition and physical function [29]. Another telemedicine intervention had positive outcomes of reduced malnutrition and improved protein intake [33]. In interventions targeting specific disease states, evidence supports “eHealth” interventions using the web and mobile-based technologies to improve diabetes care [34], and an 8-week program to increase calcium intake and exercise to improve bone health [35].

Therefore, to test the impact of a technology-based intervention on the primary outcomes of food security and diet quality, we hypothesize that older adult participants will report improved overall nutrition quality and a reduction in food security (Aim 1). To determine the effect of the intervention on secondary outcomes of technology knowledge and usage, physical activity, social isolation, and loneliness, we hypothesize that providing access and training to technologically disadvantaged older adults will increase accessibility, knowledge, and improved attitudes toward technology use (Aim 2). To examine the long-term impact and sustainability of technology use on food security, diet quality, physical activity, and social isolation, we will evaluate the sustainability of online programming integrated into the CMP, assessed via surveys and usage data (Aim 3).

## Methods

### Design

**Figure 1.**
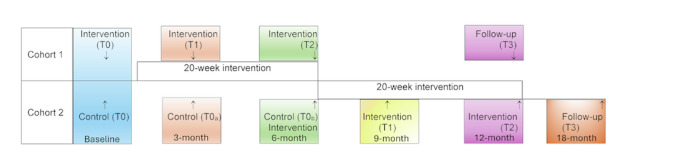
Stepped wedge cluster randomized trial schema depicting the staggered introduction of the intervention across clusters over time.

This study is a closed cohort stepped wedge design in which the timing of the intervention is randomized by cluster, with the sequential crossover of clusters from control to intervention until all clusters have received the intervention. With this design, each cluster contributes data when they are participating in the intervention and when they are not. Stepped wedge designs are useful for several reasons. First, they allow all participants to receive the intervention, and each intervention arm can serve as its control, which decreases the number of units needed. Second, the design is practical in a situation with limited resources where the intervention logistically cannot be carried out all at once. Third, the design allows researchers to examine any effects of time on the intervention’s effectiveness [36,37]. Baseline data on current dietary intake, food security, malnutrition, technology use and attitudes, physical activity, social isolation, loneliness, and demographics were collected from all participants at Time 0. Each of the 12 centers was randomly assigned to one of 2 cohorts. Participants in Cohort 1 received the intervention first. Once Cohort 1 participants finished the intervention, a crossover occurred as Cohort 2 participants began the intervention. At the 12-month time point, the second cohort completed the intervention. Data will be collected for both groups each time point from T0 to T3 (Figure 1).

### Recruitment and Procedures

#### Study Setting and Sample

Fifty-two congregate meal sites associated with the Department of Health Senior Services in San Antonio serve approximately 2400 older adults, 60 years or older in San Antonio. The sites vary by geographical location, services offered, number and demographics of older adults served, and setting. The comprehensive city-provided centers include meal service, exercise rooms and equipment, classes of varying topics from nutrition to crafts to technology, social activities, and other supportive services related to health care needs. Early in the pandemic, the congregate meal sites condensed to 11 main “hubs” that provided frozen meals twice per week to nearly 2000 older adults. Activity booklets distributed with the meals monthly provide nutritional educational materials, but this does not replace the lack of curriculum and resources normally available. San Antonio CMP participants are 73 years old, 63% female, and 70% Hispanic. Among them, 33% are categorized as impoverished based on national poverty income levels, and 15% are considered at high nutritional risk based on the DETERMINE (Disease, Eating Poorly, Tooth Loss, Economic Hardship, Reduced Social Contact, Multiple Medications, Involuntary Weight Loss, Needs Assistance, and Elderly Status) your risk nutritional screen, used to assess nutrition risk for all OAA nutrition services programs in Texas.

Participants were recruited from a total of 14 CMP sites. Flyers were used to advertise the study and information sessions. The research team conducted a comprehensive information session at each site, using a presentation describing the study’s purpose, research methods, specific data to be collected, and reviewing the consent form. Interested participants signed the consent documents, completed screening forms, and received a US$5 grocery store gift card as a token of appreciation for their time and interest. Research staff were available during the screening process to answer questions and assist older adults as needed. This multifaceted recruitment approach proved effective in ensuring thorough participant engagement and enrollment.

#### Inclusion and Exclusion Criteria

Older adults with at least one of the following: (1) inadequate or no working technology device (computer, smartphone, or tablet), (2) no or poor internet connectivity at home, and (3) lack of knowledge and usage of technology AND at least one of the following (1) food insecurity or (2) low diet quality, were considered eligible for inclusion in the study. Technical inclusion criteria were measured by questionnaires adapted from the Attitudes Toward Computers Questionnaire and technology use questionnaire from the National Health and Aging Trends Study (NHATS). Food security was assessed using the 10-question US Adult Food Security Survey Module and diet quality was screened using the Dietary Screening Tool [38]. Older adults were excluded from the study if they had a terminal disease or illness, a diagnosis of dementia or Alzheimer disease, or were unable to read or write in English or Spanish.

#### Randomization and Allocation

Twelve out of the 14 centers participated in the study and were stratified by congregate meal participant enrollment and attendance as large (>100 participants), medium (50‐100 participants), and small (<100 participants) and randomly assigned to one of 2 start times: immediately (Cohort 1) or 6 months later (Cohort 2). Site randomization by a statistician occurred after participant recruitment; however, the treatment condition was not revealed to the participants. Neither the study participants nor the study staff were blinded to the treatment conditions.

#### Participant Retention and Remuneration

Participants received compensation of a US $20 local grocery store gift card for each data collection event (T0, T1, T2, and T3). Additional rewards were built into the intervention to encourage participation and retention. Cohort 2 participants served as the active control group when not receiving the intervention. The active control consisted of monthly presentations on wellness topics not related to nutrition and bingo games led by our research team, in which they could win a US $5 local grocery store gift card. Furthermore, voluntary peer navigators, who were participants enrolled in the study and attending the center, were recruited from most sites to assist any participant requiring extra help with Zoom (Zoom Video Communications, Inc) session login, survey completion, or troubleshooting challenges within their capacity.

### Intervention Design

The City of San Antonio Senior Services Division, which oversees the CMP in Bexar County, along with Older Adults Technology Services (OATS) and the University of Texas at San Antonio (UTSA), researchers with expertise in nutrition and physical activity devised the Digital Nutrition Education Intervention. The intervention included a 5-week technology intervention, designed to address defining features of the digital divide such as access, knowledge, and usage. The 15-week online nutrition course aimed to improve food security and increase overall diet quality, while providing accurate information about nutrition and physical activity through virtual sessions.

### Formative Work

A pilot study (5 weeks) was conducted in June and July 2022 with 4 older adults who attended a local community center located in San Antonio and completed a technology class with OATS in May 2022. The community center does not have a congregate meal program. All baseline and posttesting measures and created the surveys for feedback were tested in the pilot study. Pilot data were used to inform the full 15-week intervention. All participants were satisfied with the nutrition topics, recipes, and resources provided, as well as the Friday lunch Zoom socials. A total of 75% were satisfied with the goal sheets and weekly surveys in Google Forms. All participants were satisfied with Google Classroom, but some preferred accessing the class materials by email. During the pilot study, only a single session was offered to participants. Based on their feedback, participants recommended that multiple sessions be provided to enhance scheduling, flexibility, and accommodate different availability. Process evaluation materials were finalized for the technology and nutrition intervention to document fidelity, knowledge gained, and participant satisfaction.

The creation of both the technology and nutrition intervention was informed by an advisory board consisting of UTSA researchers, key Department of Human Services (DHS) and OATS staff, and focus groups consisting of older adult CMP participants. Researchers conducted 14 focus groups with 109 CMP participants in Spring 2022 to determine the choice of technology device and nutrition topics to be incorporated into the nutrition intervention plan. Participants preferred iPads (Apple Inc) over LG tablets due to their big screen, brightness, and user-friendliness. The focus group informed the nutrition topics covered: food security and support resources, fruits and vegetables, whole grains, protein, lean meats, low-fat dairy, healthy snacks, hydration, sodium, healthy eating on a budget, reading food labels, dietary supplements, and food safety. Chronic disease-specific topics were not covered and were linked to the prevention and management of nutrition-related chronic conditions. Special emphasis on food security and the Supplemental Food Assistance Program, and other assistance programs that may help alleviate the cost of food. Three out of 5 older adults are eligible for assistance programs and do not apply for benefits [39]. Additional assistance was provided if food access was an issue. Many grocery stores and food delivery services require digital apps or online ordering, which was covered during the intervention.

### Technology Intervention

OATS is a nonprofit organization established in 2004 with a mission to help older adults live better in the digital age. OATS focuses on key areas of financial security, health and wellness, civic engagement and advocacy, social engagement, creative expression, and lifelong learning. Based in New York, OATS expanded to 6 states, including Texas in 2018, with over 26 locations. OATS has served over 30,000 older adults and has engaged San Antonio’s Mayor in the Aging Connected pledge to have 1 million seniors engage online. After participation in technology services, OATS reported 76% of participants felt more connected, 39% reported better health, 44% lost weight, 86% felt less lonely, and 94% felt more connected to the world around them. OATS has over 70 online courses and 45 online videos to support its programming.

The technology intervention addresses three key elements of the digital divide: (1) access to the internet and digital devices; (2) digital literacy or skills; and (3) internet usage and value. Access and use of the internet were reported by 67% of adults 65 years or older in the United States [19]. Although internet usage in older adults has increased significantly over the past 5 years [40], digital inequalities and disparities exist for at-risk populations. Digital inequalities can further describe the digital divide in terms of usage, skills, social support, and self-perception. Older adults who use the internet could have limitations in skill, social support, and attitudes that can further describe passive participation in our digital society [41]. Disparities in racial or ethnic minorities further expand the digital divide and are compounded by low socioeconomic status [42,43].

Using OATS’ expertise in providing technology education and services, enrolled participants received a technology bundle. The technology bundle included a tablet computer device selected by the focus groups and covers 1 year of internet connectivity and technical support provided by OATS. The participants attended a 5-week iPad course that provides information on iPad essentials, introduction to digital culture, and physical activity resources in small groups of less than 15 in person at the CMP sites. In addition, peer navigators (participants who identified as being comfortable in technology skills) were recruited from CMP sites to assist those struggling with technology and having trouble completing weekly tasks in Google Classroom.

### Nutrition Intervention

**Figure 2.**
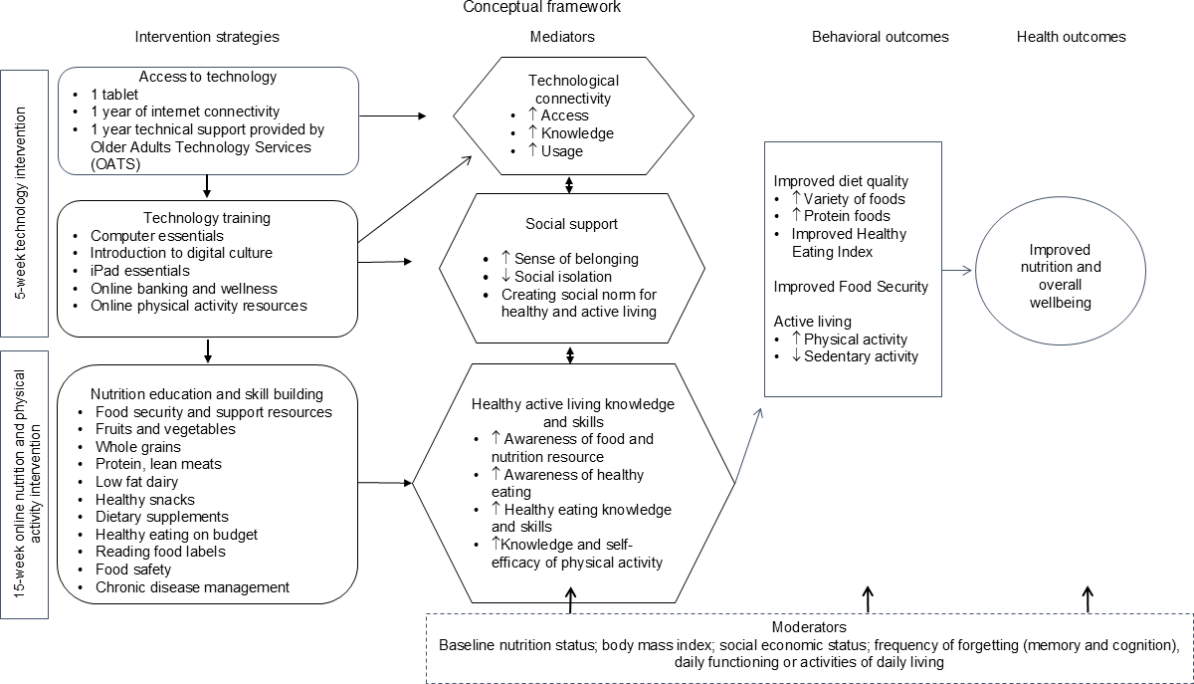
Conceptual framework outlining the digital nutrition study design and key components.

The nutrition intervention aims to restore and enhance nutrition programming for congregate meal participants online after bridging digital connectivity, access to devices, and the applied use of technology. Key intervention strategies to address technology and nutrition will be used to improve diet quality, food security, physical activity, technology use, and socialization (Figure 2). Long-term maintenance of the online curriculum will be managed by DHS Senior Services program, and the sustainability of the project will be measured after 6 months.

Suggestions from the literature recommend soliciting older adult participation in the technical intervention design and considering barriers to technological intervention [31]. Our nutrition intervention was developed in coordination with input from a subset of older adult CMP participants and the registered dietitian from DHS Senior Services. In addition, we designed the intervention based on successful dietary behavioral change interventions and successful digital interventions with older adults by the following: defined and clear topics relevant to older adult health and the population, using proven behavioral theories, using incentives, and sufficient contact. The intervention was informed by the Social Cognitive Theory that addresses intrapersonal and interpersonal influences on behavior change. Weekly goal setting and goal evaluation were incorporated to increase participants’ self-efficacy in eating healthy. Social support systems embedded in the intervention encouraged and rewarded behavior changes. All materials were created at a sixth-grade reading level or below and made available in English and Spanish. Only 1‐2 learning objectives for each topic were created. A digital reward-based program was created to provide incentives for participation and behavior change. Rewards were in the form of digital “badges” like what is used in online sites and applications for achievements [44,45]. The benefits of this system include a no-cost reward system that may motivate participation and signify progress in learning [45,46]. Weekly and monthly rewards were given for attendance and completing weekly tasks which were captured in a digital format. Graduates of the program were given a certificate at a graduation ceremony conducted at the centers in person.

Weekly modules in Google Classroom included nutrition, Microsoft PowerPoint presentations and supplementary resources, handouts, videos, and recipes to enhance learning and practical application. Physical activity resources such as prerecorded (<10 min) classes and resources, videos, and handouts were included to complement the nutrition intervention. The physical activity classes were designed to cater to the specific needs and abilities of older adults, including low-impact activities designed to be gentle on the joints and help improve cardiovascular health, strength, balance, and flexibility. The physical activity classes also provided information on the importance of nutrition and how it can enhance physical performance and recovery.

All sessions were conducted virtually using the Zoom videoconferencing platform. Email reminders for Zoom sessions and links were sent through Google Classroom and provided in Google Calendar. Each week, 3 sessions were conducted in English at different times of the day, and one session was conducted in Spanish, and participants were encouraged to attend live sessions conducted by a registered dietitian or a supervised dietetic student to provide sufficient contact and flexibility. Each session lasted between 40 minutes and an hour and included a Microsoft PowerPoint presentation based on the predetermined weekly topics (Table 1). Time was allocated at the end of each interactive session for questions and answers. One English session and 1 Spanish session were recorded and sent to all participants midway through the week for viewing if they missed attending the live session or if they would like to rewatch.

**Table 1.** Specific topics, learning objectives, clear messaging, and rewards.

Week	Topic	Learning objectives	Clear messaging	Rewards^a^
1	Introduction	Use the tablet to access live and recorded sessions. List possible rewards. Identify ways to document participation.	Document participation and create goals each week. Participate in 4 out of 5 activities per week to receive rewards.	Digital reward Content Attendance
2	Nutrition and Physical Activity for Healthy Aging	Define healthy aging. List ways nutrition and physical activity can prevent or help manage certain diseases. List the food groups of MyPlate.	Good nutrition and regular physical activity can positively impact overall health. MyPlate meals can help you eat healthy foods.	Digital reward Content Attendance
3	Food security and support resources	Define food security. Identify resources that can help with food insecurity. Assess qualifications for any resources to address food security.	Use all the resources you qualify for to help with any food insecurity.	Digital reward Content Attendance
4	Reading food labels	Identify sections of the food label. Identify nutrients that are high or low in a food product.	Reading labels can identify healthy foods.	Digital reward Content Attendance
5	Fruits and vegetables	Describe the health benefits of fruits and vegetables. List the fruit and vegetable recommendations for older adults. Identify fruit and vegetable servings.	Eat a rainbow of fruits and vegetables to gain a variety of nutrients. Eat 2 servings of fruit and 3 servings of vegetables to gain maximum health benefits.	Digital reward Content Attendance
6	Carbohydrates and grains	List foods that contain carbohydrates. Describe the difference between whole and refined grains. Describe how foods with carbohydrates can affect my body.	Many foods contain carbohydrates that are needed to fuel your body. Make half your grains whole.	Digital reward Content Attendance
7	Fats	List foods that contain fats. Describe the health benefits of fats. List fats we need to limit or avoid in our diet.	Healthy fats in our diet can help reduce the risk of certain diseases. Limiting saturated fat and cholesterol is important for a healthy diet.	Digital reward Content Attendance
8	Protein	List foods high in protein. Describe why dietary protein is important for healthy aging. List the recommendations for protein for older adults.	Including adequate protein in our diet can help maintain health. Protein foods come from both plant and animal sources.	Digital reward Content Attendance
9	Season food without salt	Define sodium. Describe how excess sodium relates to health. Describe ways to flavor food without salt.	Flavoring foods without salt can reduce excess sodium intake in the diet.	Digital reward Content Attendance
10	Beverages	Identify healthy beverage options. Describe why hydration is important for older adults.	Consuming beverages helps prevent dehydration. Healthy beverages contain little to no sugar.	Digital reward Content Attendance
11	Healthy snacks	Describe the importance of consuming healthy snacks for older adults. List 3 ways to make healthy snacks.	Healthy snacks can help provide important nutrients in our diet. Healthy snacks contain 2 or more food groups.	Digital reward Content Attendance
12	Eating healthy on a budget	Describe how planning meals helps optimize grocery store visits. List inexpensive easy ways to eat healthy.	Eating healthly on a budget is possible with adequate planning.	Digital reward Content Attendance
13	Dietary supplements	Define dietary supplements. Describe why older adults may need to consume dietary supplements. List ways dietary supplements may cause harm.	Dietary supplements may be recommended in certain situations. Dietary supplements can harm our health if not used appropriately.	Digital reward Content Attendance
14	Food safety	Define foodborne illness. Describe why older adults are more at-risk for foodborne illness. Describe how to prevent foodborne illness.	Older adults are at increased risk for foodborne illnesses. Food may cause illness if not prepared, cooked, or stored properly.	Digital reward Content Attendance
15	Celebration	Celebrate goals achieved and knowledge gained.	Making small changes can improve nutrition choices and healthy habits.	Digital reward Content Attendance Celebration

a Mapping of specific topics to corresponding learning objectives, with emphasis on clear messaging strategies and reinforcement through rewards, developed to support the Digital Nutrition Intervention study.

Participants were required to complete a weekly goal-setting survey, a goal check-in survey, and a week-specific survey. An optional satisfaction survey was sent each week to gather feedback. In addition, optional weekly Zoom social sessions hosted by dietetic and public health students were offered twice a week to facilitate social interaction in the digital space. This was mutually beneficial for the students and the older adults in providing cross-generational interactions to facilitate a greater understanding and empathy for other generations [47]. Older adults may also have added health benefits from this type of interaction [48]. Dietetic students benefited from the practical training experience, while older adults benefited from the socialization.

### Measurements

Table 2 lists all the measurements incorporated in the study.

**Table 2.** Overview of measurements applied in the digital nutrition intervention study.

Measurement		Data collection timeline	Outcomes
Diet Quality Score		Measured at all data collection timepoints.	Developed to assess diet quality and nutrition risk [38]. Completed within 15 minutes and used to assess the Diet Quality Score [49]. Score based on overall diet quality (greater than 75=not at risk, between 60‐75=possible risk, less than 60=at risk) [50].
Healthy Eating Index (ASA24 Food Recall)		Prior, during, and after the assigned cohort intervention.	Measurement of dietary quality on recommendations from the Dietary Guidelines for Americans [51]. Completed using the Automated Self-administered 24-Hour Dietary Assessment (ASA24) online tool developed by the National Cancer Institute [52]. Research team will assist participants in the completion of the ASA24 food recall. Scoring is on a scale of 1 to 100 for the following food groups: fruits, vegetables, greens, whole grains, dairy, protein, seafood, plant proteins, fatty acids, refined grains, sodium, added sugars, and saturated fats [51].
Food security		Measured at all data collection timepoints.	Helps to determine financial barriers for safe access to food in the last 3 months [53]. The 10-question US Adults Food Security Survey Module will be used to assess food security in the following categories and score: 0=high food security, 1‐2=marginal food security, 3‐5=low food security, ≥6=very low food security [54].
Physical Activity Scale for the Elderly (PASE**)**		Measured at all data collection timepoints.	Used to assess self-reported physical activity in community-dwelling older adults over 7 days by adults aged 65 years and above [55]. PASE scores range from 0‐400 or more and are closely associated with grip strength and balance [55].
Short Physical Performance Battery (SPPB)		Prior, during, and after the assigned cohort intervention.	Developed by the National Institute of Aging to assess lower extremity functioning in older adults. SPPB includes gait speed, chair stand, and balance tests [56]. Overall scores can range from 0 (worst performance) to 12 (best performance) and are predictive of risk for frailty, mortality, nursing home admission, and disability [57].
Attitudes Toward Computers Questionnaire (ATCQ)		Measured at all data collection timepoints.	9-question Technology Attitudes Survey is used to measure a person’s comfort and interest in tablet computers [43,58,59]. Participants complete a 20-question survey rated on a 5-point Likert scale (strongly agree to strongly disagree). The final score is computed by adding scores from all questions.
Technology use questionnaire from the National Health and Aging Trends Study (NHATS)		Measured at all data collection timepoints.	9-question Technology Access and Usage Survey is used to measure technology comfort, typical daily use, and access in older adults [60]. Overall scores range from 0 to 9. A score of 0‐1 is classified as poor technology access and usage, scores of 2‐5 indicate moderate access and usage, and a score of 6‐9 signifies good technology access and usage.
OATS^a^ technology survey		Administered before, during, and after the 5-week technology essentials class.	Internal satisfaction survey following the completion of the 5-week technology essentials course administered by OATS. The data obtained from this survey will be used to evaluate the participants’ technology proficiency before and after the course, as well as to measure their satisfaction with the course materials, content, level of difficulty, and the instructor.
Social Disconnectedness Scale and Perceived Isolation Scale		Measured at all data collection timepoints.	Scale measures the degree of social isolation using two scales. The Social Disconnectedness Scale includes 13 items including restricted social networks and social inactivity [61]. The Perceived Isolation Scale includes 10 items including lack of support and loneliness [61].
3-item UCLA^b^ Loneliness Scale		Measured at all data collection timepoints.	The questionnaire assesses the degree of perceived loneliness using the 3-item UCLA Loneliness Survey (PhenX Toolkit) [62].
Malnutrition – Mini Nutrition Assessment (MNA)		Measured at baseline, 6 months, and 6-month postintervention.	5-question Mini Nutrition Assessment Short Form (MNA-SF) will be used as a screening tool to identify older adults who are malnourished or at risk for malnutrition [63]. Score greater than or equal to 12 indicates normal nutritional status, a score between 11 and 8 suggests being at risk of malnutrition, and a score less than or equal to 7 indicates malnourishment [64].
Frequency of Forgetting questionnaire		Measured at baseline, 6 months, and 6-month postintervention.	Assesses subjective memory impairments in older adult participants [65]. Scoring is based on the following categories: major problems, some minor problems, and no problems.
Activities of daily living questionnaire		Measured at baseline, 6 months, and 6-month postintervention.	The PhenX Toolkit was used to assess activities of daily living at baseline, 6 months, and 12 months [66]. Participants completed a 16-question survey and ability to complete activities: bathing, grooming, dressing upper body, dressing lower body, feeding, using toilet, walking, and getting in/out of chair.
Height, weight, and BMI		Measured at baseline, 6 months, and 6-month postintervention.	Measured without shoes and with lightweight clothing using a calibrated scale (Seca 876) and portable stadiometer (Seca 213). BMI will be calculated from the measured height and weight and will be used as part of the MNA score calculation. BMI categories are classified as follows: BMI less than 19=0, BMI 19‐20.9=1, BMI 21‐22.9=2, and BMI 23 or above =3 [67].
Demographic information and medical history		Gathered during screening.	Gathered responses on age, sex, residence zip code, senior center attended, race/ethnicity, who lives in the household, how many people live in the household, annual household income, education (highest level attended), current employment status, history of smoking, whether receiving benefits such as social security, disability, Supplemental Food Assistance Program (SNAP) or retirement benefits, diagnosis of medical conditions, and medication list.

a OATS: Older Adults Technology Services.

b UCLA: University of California, Los Angeles

### Process Evaluation

The process evaluation design is informed by the National Institutes of Health Behavior Change Consortium Treatment Fidelity Workgroup’s best practice recommendations [68]. Multiple indicators will be used to evaluate the fidelity and completeness of the implementation of all intervention components and determine the contribution of each component to the primary and secondary outcomes. All process evaluation measures present a minimum participant burden. Three major aspects of process evaluation will be measured. First, the intervention dose delivered (ie, the extent to which the technology and nutrition intervention is delivered as planned to older adult participants) will be measured by: (1) technology use records that will measure the times the participant interacted with the intervention materials; (2) completion of delivery schedule from OATS staff to indicate technology equipment and support has been delivered to the participants; (3) evaluation of staff training; and (4) monthly auditing of intervention lesson delivery. Second, the intervention dose received (ie, the extent to which the older adult participants understand and learn the technology and nutrition knowledge and skills delivered in the intervention) will be assessed by: (1) attendance records collected when older adults log in to the intervention site; (2) pre and post knowledge test after each intervention activity; and (3) poststudy focus groups on program delivery process and feedback with intervention staff (n=10). Third, participants’ responses to the intervention (ie, the extent to which the older adult participants use and apply the knowledge and skills learned in the intervention in daily life) will be evaluated by: (1) monthly surveys to gather feedback from older adults on their use and satisfaction with the intervention materials; and (2) poststudy focus groups with a subgroup of participants to assess their satisfaction with intervention components/activities and ideas for improving the intervention in the future.

### Power Calculations and Estimation of Sample Size

Our sample size calculation was conducted based on examining the effect sizes of similar studies in the current literature [68]. Effect sizes for fruit and vegetable intake ranged from 0.41 to 0.8 [9,28]. Using the smaller effect sizes and considering the stepped wedge design as a clustered randomized trial, with an Interrelation Class Coefficient of 0.016 [37] estimated from the literature (thereby reducing our effective sample size), a total of 210 participants (105 per group) is required to reach 80% power. With an expected N*=*410 participants in the analytic sample (effective N=374), we will be able to detect an effect size of 0.30 with 80% power and 97.7% power to detect an effect of 0.41 [69]. To detect a difference with an effect size of 0.3 or greater with 80% power at an alpha level of 0.05, 352 participants are required in the analytic sample. Power was calculated using the stepped wedge command in STATA/SE (12.1; StataCorp LLC). The details are described in Hemming and Girling [36].

### Data Analysis

#### Baseline Equivalency

Baseline characteristics for each cohort at the individual and cluster level will be used to determine if any imbalances exist in either demographic variable or baseline dietary intake and food security. Effect sizes (ES) for differences between cohorts will be assessed with Hedges *g* (for continuous variables) and Cox index (for dichotomous variables). Any variables with effect size between .05 and .25 will be added to models to adjust for pre-existing differences.

#### Missing Data

Missing data will be imputed using full information maximum likelihood. As with most longitudinal studies, participants leave the study at different times, with attrition commonly increasing over time. Traditional methods of handling missing data require the researcher to discard data or impute unknown values, which frequently causes estimation bias and loss of statistical power. These missing data concerns are ameliorated when using full information maximum likelihood, as it does not have the same limitations as other more common approaches (eg, listwise deletion and regression imputation methods).

#### Statistical Models

##### Aim 1

Primary outcome data will be analyzed in Aim 1. For each of the 3 outcomes in Aim 1 (Dietary Quality Score and Food Security), at 3-month and 6-month periods, a 2-level multilevel model (MLM) will be constructed that examines change over time for individual participants within centers. MLMs consider that participants within a cluster (eg, a center) share characteristics that make them more like each other than participants at other centers. MLM has several distinct advantages over other modeling strategies: (1) MLM has greater statistical power given that the estimated standard errors are corrected for model dependency; (2) there is no need to assume or test for the homoscedasticity or sphericity assumptions, as these variance and covariance components are estimated within the MLM model. Depending on model complexity and whether the statistical assumptions are met, the models will be estimated via Bayesian estimation or maximum likelihood estimation with robust standard errors. For Level 1 (participant level), the outcome variable of interest will be modeled using pretest score on the outcome measure, treatment status (ie, control or experimental), and any nonequivalent baseline participant characteristics as covariates. Level 2 (center level) will model the cluster effect of centers on outcomes.

##### Aim 2

Data collected for Aim 2 represent our secondary research questions (eg, physical activity, technology knowledge and attitudes, social isolation, and loneliness). Data collected at all time points (Physical Activity Scale for the Elderly [PASE], technology knowledge and attitudes, social isolation, and loneliness) will be analyzed using MLM described in Aim 1. To minimize participant fatigue, data for this secondary research aim will be collected at defined intervals (T0, T1, and T2). These data will include the Healthy Eating Index and Short Physical Performance Battery (SPPB) to assess physical function. Differences between groups will be assessed at T2 for each outcome via 2-level covariate MLM models. Level 1 (participant level) will test whether treatment status (ie, control or experimental), pretest score on dietary quality, and food security, and any nonequivalent baseline participant characteristics predict outcomes. At Level 2 (center level), we will model the influence of the 7 centers.

##### Aim 3

To assess the long-term impacts of the program at T3, Level 1 (time) will model the change in the outcome variables throughout the study (T0, T1, T2, and T3) within each participant. Specifically, each participant’s random intercept (eg, initial status) and the random slope over the 3 time periods will be calculated, and the time variable will represent the change in a particular outcome variable (ie, food security) over time within each participant. Level 2 (time nested within participant characteristics) will test whether treatment status (ie, control or experimental) and any nonequivalent baseline participant characteristics predict outcomes. If the ICC coefficient is greater than 0.10, a third level will be added to the model to control for clustering by center. Finally, participants will be clustered within centers (Level 3).

### Ethical Considerations

This study is approved by the University of Texas at San Antonio Institutional Review Board (IRB protocol FY20-21-241). The baseline phase of the study involved obtaining institutional review board approval through a comprehensive review, followed by participant recruitment and baseline data collection. During this phase, participants also reviewed and signed consent forms in writing.

Data are collected using Research Electronic Data Capture (REDCap; Vanderbilt University) surveys and are managed and stored in REDCap and in a UTSA-secured site where only the study team has access to participant files. REDCap is a secure, web-based software platform designed to support data capture for research studies, providing (1) an intuitive interface for validated data capture; (2) audit trails for tracking data manipulation and export procedures; (3) automated export procedures for seamless data downloads to common statistical packages; and (4) procedures for data integration and interoperability with external sources [70,71]. All data and records generated during this study comply with confidentiality procedures and protocols in accordance with UTSA and Health Insurance Portability and Accountability Act (HIPAA) policies. We will not share any identifiable data with outside study partners unless specified in the consent form. Data collection is the responsibility of the research study staff at the site under the supervision of the site investigator.

Participants receive a compensation of US $20 local grocery store gift card for each data collection event and the opportunity to win a US $5 local grocery store gift card during the bingo games.

## Results

**Figure 3.**
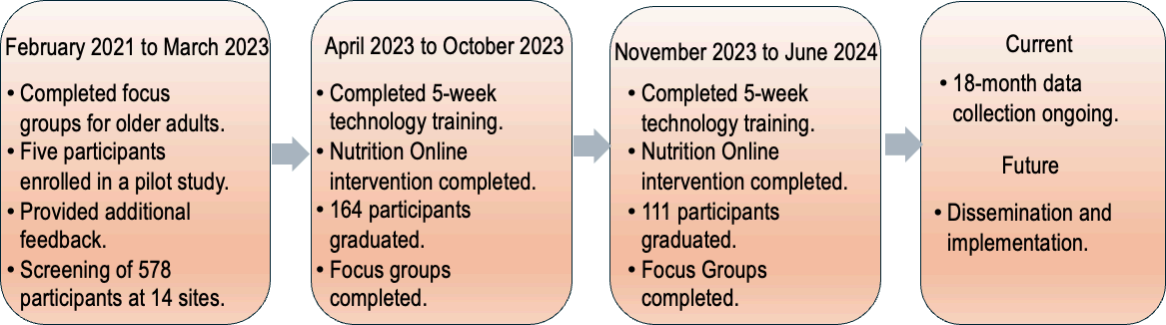
Study progress.

The project was funded in October 2022. As indicated in the study progress (Figure 3), recruitment and screening started in June 2022. The research team recruited and screened 150 participants by the end of August 2022 at the 7 original target centers. Due to the low recruitment numbers, the number of centers and recruitment time were extended. Seven additional centers for recruitment were incorporated starting in September 2022. Power analysis was completed to ensure adequate recruitment numbers were met with the 14 centers. NIH was notified in writing of these changes. Fourteen centers were condensed to 12 centers (1 closed because of structural damage and 1 smaller center merged with a larger sister center).

**Figure 4.**
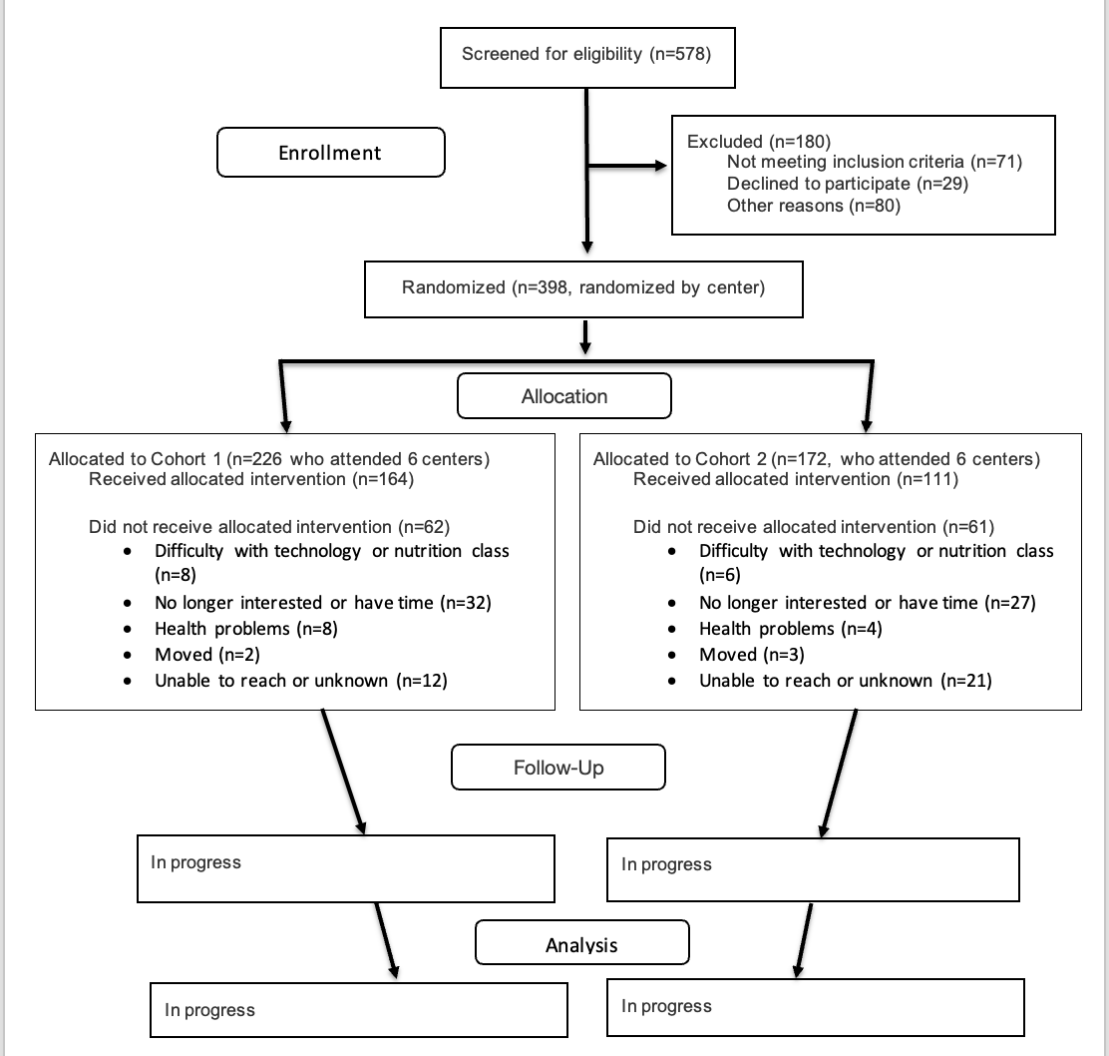
Consolidated Standards of Reporting Trials (CONSORT) flow diagram.

A total of 578 older adults completed the screening, of whom 507 participants were eligible to participate in the study (88%). The 12 centers were randomly assigned into 2 study cohorts, which consisted of a total of 398 older adult participants. Cohort 1 consisted of 226 eligible participants, of whom 164 participants graduated from the program. Cohort 2 had 172 eligible participants, of whom 111 participants graduated from the program (Figure 4). Monthly bingo and informative presentations (nonnutrition and nonphysical activity–related) were conducted with Cohort 2 centers for engagement and retention. Focus groups were conducted for Cohort 1 postintervention in October 2023 and for Cohort 2 in June 2024 to assess the fidelity of the intervention. Data collection for Cohort 2 is currently ongoing. Preliminary data analysis is being conducted for all cohorts to evaluate the intervention’s outcomes.

## Discussion

### Principal Considerations

Inadequate nutrition and a lack of physical activity can lead to complications from chronic diseases in older adults and a decline in health [72,73]. The COVID-19 pandemic disrupted essential nutrition services like CMP sites in San Antonio, Texas [18]. The “digital divide” exacerbated the negative impact of the COVID-19 pandemic on older adults [18]. To address this, improving technology access and knowledge, and providing evidence-based online nutrition programs are essential and have the potential to benefit the vulnerable homebound population, thereby becoming an integral part of OAA-funded services.   

### Strengths and Limitations

The program’s strengths lie in the potential for its integration into the existing CMP infrastructure, bolstering support and resources for participants most in need. The proposed intervention follows a community-based participatory research approach, seeking essential input and feedback from the DHS Senior Services, OATS, and older adult CMP participants to enhance the intervention’s chances of success [74]. Seeking the assistance of the voluntary peer navigators at the centers provided additional in-person support to participants experiencing challenges in completing the intervention. The technology intervention addresses key barriers to older adults adopting technology by providing internet services and devices, offering support through a 5-week training program conducted by OATS, and providing continued technical support and internet access for one year [14,41]. In addition, OATS also provided practice Zoom sessions and in-person help to improve participant self-efficacy in navigating through the Zoom sessions and the nutrition modules in Google Classroom. The DHS team acted as a medium of communication between the study team and the participants.

A second strength of the study is the closed-cohort stepped-wedge design. In this approach, cohorts are randomly assigned the timing of the intervention, with a sequential transition from control to intervention for each cohort until all participants have received the intervention. This design ensures that all participants benefit from the intervention and allows each group to serve as its own control. It is practical when resources are limited and allows for the analysis of the influence of time on the intervention’s effectiveness.

Given the increased reliance on technology post-COVID-19, offering a digital nutrition intervention has the potential to positively impact food security and diet quality among vulnerable populations [75]. Moreover, this project has the potential to enhance physical activity, improve socialization, and mitigate loneliness in an at-risk, predominantly Hispanic population in South Texas.

Although the proposed intervention offers technology training, support, and access to the internet and devices, some limitations exist. A few participants may continue to experience challenges in understanding and using technology to engage in the online nutrition sessions conducted via Zoom. Moreover, the technical support and internet access provided by OATS ceased after the one-year service period, thereby restricting participants from using their devices except at the CMP site or locations offering complimentary Wi-Fi connectivity. Furthermore, the accuracy of survey completion may be affected by recall bias and introspective ability. To enhance the long-term sustainability of the project, mitigate associated costs, and strengthen the scope of incorporation into OAA services, it is important to investigate alternative options that offer low-cost internet access and affordable devices.

### Conclusions

This intervention study, conducted in collaboration with community partners and older adults, is focused on delivering a culturally tailored digital nutrition curriculum to at-risk older adults who frequently attend congregate meal sites. This initiative followed the allocation of technological resources and requisite support. The study’s developed materials are designed for implementation across all senior center locations in San Antonio. If successful, the project has the potential to reach over 2000 older adult congregate meal participants throughout San Antonio. Furthermore, it could serve as a replicable model for other OAA-funded nutrition services. The study’s outcomes will be disseminated through publication in high-impact journals and presentations at regional and national nutrition conferences dedicated to nutrition and aging.

## Supplementary material

10.2196/65976 Checklist 1 CONSORT-eHEALTH checklist (V 1.6.1).

## Abbreviations

CMP: congreate meal program
DETERMINE: Disease, Eating Poorly, Tooth Loss, Economic Hardship, Reduced Social Contact, Multiple Medications, Involuntary Weight Loss, Needs Assistance, and Elderly Status
DHS: Department of Human Service
HIPAA: Health Insurance Portability and Accountability Act
MLM: multilevel model
NHATS: National Health and Aging Trends Study
OAA: Older Americans Act
OATS: Older Adults Technology Services
REDCap: Research Electronic Data Capture
UTSA: University of Texas at San Antonio

## References

[1] (2020). Profile of older americans. https://acl.gov/aging-and-disability-in-america/data-and-research/profile-older-americans.

[2] Topinková E (2008). Aging, disability and frailty. Ann Nutr Metab.

[3] Amarantos E, Martinez A, Dwyer J (2001). Nutrition and quality of life in older adults. J Gerontol A Biol Sci Med Sci.

[4] Gkiouras K, Cheristanidis S, Papailia TD (2020). Malnutrition and food insecurity might pose a double burden for older adults. Nutrients.

[5] (2014). Food insecurity among older adults. AARP.

[6] Bollwein J, Volkert D, Diekmann R (2013). Nutritional status according to the mini nutritional assessment (MNA®) and frailty in community dwelling older persons: a close relationship. J Nutr Health Aging.

[7] van den Helder J, Mehra S, van Dronkelaar C (2020). Blended home-based exercise and dietary protein in community-dwelling older adults: a cluster randomized controlled trial. J Cachexia Sarcopenia Muscle.

[8] Verlaan S, Ligthart-Melis GC, Wijers SLJ, Cederholm T, Maier AB, de van der Schueren MAE (2017). High prevalence of physical frailty among community-dwelling malnourished older adults-a systematic review and meta-analysis. J Am Med Dir Assoc.

[9] Wei K, Nyunt MSZ, Gao Q, Wee SL, Ng TP (2019). Long-term changes in nutritional status are associated with functional and mortality outcomes among community-living older adults. Nutrition.

[10] Saffel-Shrier S, Johnson MA, Francis SL (2019). Position of the academy of nutrition and dietetics and the society for nutrition education and behavior: food and nutrition programs for community-residing older adults. J Nutr Educ Behav.

[11] Balistreri KS (2022). Older adults and the food security infrastructure. Appl Econ Perspect Policy.

[12] Mabli J, Shenk M (2021). Continued participation in congregate meal programs: the role of geographic access to food. J Nutr Gerontol Geriatr.

[13] Wu YH, Damnée S, Kerhervé H, Ware C, Rigaud AS (2015). Bridging the digital divide in older adults: a study from an initiative to inform older adults about new technologies. Clin Interv Aging.

[14] Tappen RM, Cooley ME, Luckmann R, Panday S (2022). Digital health information disparities in older adults: a mixed methods study. J Racial Ethn Health Disparities.

[15] Choukou MA, Sanchez-Ramirez DC, Pol M, Uddin M, Monnin C, Syed-Abdul S (2022). COVID-19 infodemic and digital health literacy in vulnerable populations: A scoping review. Digit Health.

[16] Morley JE, Vellas B (2020). Editorial: COVID-19 and older adults. J Nutr Health Aging.

[17] Brooke J, Jackson D (2020). Older people and COVID-19: isolation, risk and ageism. J Clin Nurs.

[18] Krendl AC, Perry BL (2021). The impact of sheltering in place during the COVID-19 pandemic on older adults’ social and mental well-being. J Gerontol B Psychol Sci Soc Sci.

[19] Morrow-Howell N, Galucia N, Swinford E (2020). Recovering from the COVID-19 pandemic: a focus on older adults. J Aging Soc Policy.

[20] Firman J, Bedlin H, Phillips M, Hodges J (2019). Strengthening innovations in aging services through the next reauthorization of the older Americans act. Public Policy Aging Rep.

[21] Stroebele-Benschop N, Depa J, de Castro JM (2016). Environmental strategies to promote food intake in older adults: a narrative review. J Nutr Gerontol Geriatr.

[22] Sahyoun NR, Pratt CA, Anderson A (2004). Evaluation of nutrition education interventions for older adults: a proposed framework. J Am Diet Assoc.

[23] Bandayrel K, Wong S (2011). Systematic literature review of randomized control trials assessing the effectiveness of nutrition interventions in community-dwelling older adults. J Nutr Educ Behav.

[24] Neves FJ, Tomita LY, Liu A, Andreoni S, Ramos LR (2020). Educational interventions on nutrition among older adults: a systematic review and meta-analysis of randomized clinical trials. Maturitas.

[25] Brewer D, Dickens E, Humphrey A, Stephenson T (2016). Increased fruit and vegetable intake among older adults participating in Kentucky’s congregate meal site program. Educ Gerontol.

[26] Hersey JC, Cates SC, Blitstein JL (2015). Eat smart, live strong intervention increases fruit and vegetable consumption among low-income older adults. J Nutr Gerontol Geriatr.

[27] Wunderlich S, Bai Y, Piemonte J (2011). Nutrition risk factors among home delivered and congregate meal participants: Need for enhancement of nutrition education and counseling among home delivered meal participants. The Journal of nutrition, health and aging.

[28] Mitchell RE, Ash SL, McClelland JW (2006). Nutrition education among low-income older adults: a randomized intervention trial in congregate nutrition sites. Health Educ Behav.

[29] van Doorn-van Atten MN, de Groot LC, Romea AC, Schwartz S, de Vries JH, Haveman-Nies A (2019). Implementation of a multicomponent telemonitoring intervention to improve nutritional status of community-dwelling older adults: a process evaluation. Public Health Nutr.

[30] van Doorn-van Atten MN, Haveman-Nies A, van Bakel MM (2018). Effects of a multi-component nutritional telemonitoring intervention on nutritional status, diet quality, physical functioning and quality of life of community-dwelling older adults. Br J Nutr.

[31] Park S, Kim B (2020). Readiness for utilizing digital intervention: patterns of internet use among older adults with diabetes. Prim Care Diabetes.

[32] Kiss N, Baguley BJ, Ball K (2019). Technology-supported self-guided nutrition and physical activity interventions for adults with cancer: systematic review. JMIR Mhealth Uhealth.

[33] Marx W, Kelly JT, Crichton M (2018). Is telehealth effective in managing malnutrition in community-dwelling older adults? A systematic review and meta-analysis. Maturitas.

[34] Rollo ME, Aguiar EJ, Williams RL (2016). eHealth technologies to support nutrition and physical activity behaviors in diabetes self-management. Diabetes Metab Syndr Obes.

[35] Nahm ES, Resnick B, Brown C (2017). The effects of an online theory-based bone health program for older adults. J Appl Gerontol.

[36] Hemming K, Haines TP, Chilton PJ, Girling AJ, Lilford RJ (2015). The stepped wedge cluster randomised trial: rationale, design, analysis, and reporting. BMJ.

[37] Mdege ND, Man MS, Taylor Nee Brown CA, Torgerson DJ (2011). Systematic review of stepped wedge cluster randomized trials shows that design is particularly used to evaluate interventions during routine implementation. J Clin Epidemiol.

[38] Bailey RL, Miller PE, Mitchell DC (2009). Dietary screening tool identifies nutritional risk in older adults. Am J Clin Nutr.

[39] (2018). 7 facts about older adults and SNAP. https://www.ncoa.org/article/7-facts-about-older-adults-and-snap/.

[40] Chiu CJ, Kuo SE, Lin DC (2019). Technology-embedded health education on nutrition for middle-aged and older adults living in the community. Glob Health Promot.

[41] Seifert A (2020). The digital exclusion of older adults during the COVID-19 pandemic. J Gerontol Soc Work.

[42] Yoon H, Jang Y, Vaughan PW, Garcia M (2020). Older Adults’ Internet Use for Health Information: Digital Divide by Race/Ethnicity and Socioeconomic Status. J Appl Gerontol.

[43] Choi NG, Dinitto DM (2013). The digital divide among low-income homebound older adults: Internet use patterns, eHealth literacy, and attitudes toward computer/Internet use. J Med Internet Res.

[44] Huffman FG, Vaccaro JA, Vieira ER, Zarini GG (2017). Health-related characteristics of older adults who attend congregate meal sites in the United States. Geriatrics (Basel).

[45] Shields R, Chugh R (2017). Digital badges – rewards for learning?. Educ Inf Technol.

[46] Vieira ER, Vaccaro JA, Zarini GG, Huffman FG (2017). Health indicators of US older adults who received or did not receive meals funded by the older Americans act. J Aging Res.

[47] June A, Andreoletti C (2020). Participation in intergenerational service-learning benefits older adults: a brief report. Gerontol Geriatr Educ.

[48] Zhong S, Lee C, Foster MJ, Bian J (2020). Intergenerational communities: a systematic literature review of intergenerational interactions and older adults’ health-related outcomes. Soc Sci Med.

[49] MacNab L, Francis SL, Lofgren I (2018). Factors Influencing Dietary Intake Frequencies and Nutritional Risk among Community-Residing Older Adults. J Nutr Gerontol Geriatr.

[50] Cox LA, Hwang S, Haines J (2021). Using the PhenX Toolkit to Select Standard Measurement Protocols for Your Research Study. Curr Protoc.

[51] Krebs-Smith SM, Pannucci TE, Subar AF (2018). Update of the Healthy Eating Index: HEI-2015. J Acad Nutr Diet.

[52] Subar AF, Kirkpatrick SI, Mittl B (2012). The Automated Self-Administered 24-hour dietary recall (ASA24): a resource for researchers, clinicians, and educators from the National Cancer Institute. J Acad Nutr Diet.

[53] Blumberg SJ, Bialostosky K, Hamilton WL, Briefel RR (1999). The effectiveness of a short form of the Household Food Security Scale. Am J Public Health.

[54] Byker Shanks C, Calloway EE, Parks CA, Yaroch AL (2020). Scaling up measurement to confront food insecurity in the USA. Transl Behav Med.

[55] Washburn RA, Smith KW, Jette AM, Janney CA (1993). The Physical Activity Scale for the Elderly (PASE): development and evaluation. J Clin Epidemiol.

[56] Guralnik JM, Simonsick EM, Ferrucci L (1994). A short physical performance battery assessing lower extremity function: association with self-reported disability and prediction of mortality and nursing home admission. J Gerontol.

[57] Rinaldo L, Caligari M, Acquati C (2022). Functional capacity assessment and Minimal Clinically Important Difference in post-acute cardiac patients: the role of Short Physical Performance Battery. Eur J Prev Cardiol.

[58] Lee CC, Czaja SJ, Moxley JH (2019). Attitudes toward computers across adulthood from 1994 to 2013. Gerontologist.

[59] Jay GM, Willis SL (1992). Influence of direct computer experience on older adults’ attitudes toward computers. J Gerontol.

[60] Kasper JD, Freedman VA (2014). Findings from the 1st round of the National Health and Aging Trends Study (NHATS): introduction to a special issue. J Gerontol B Psychol Sci Soc Sci.

[61] Cornwell EY, Waite LJ (2009). Measuring social isolation among older adults using multiple indicators from the NSHAP study. J Gerontol B Psychol Sci Soc Sci.

[62] Hughes ME, Waite LJ, Hawkley LC, Cacioppo JT (2004). A short scale for measuring loneliness in large surveys: results from two population-based studies. Res Aging.

[63] Hamirudin AH, Charlton K, Walton K (2016). Outcomes related to nutrition screening in community living older adults: A systematic literature review. Arch Gerontol Geriatr.

[64] Vellas B, Guigoz Y, Garry PJ (1999). The Mini Nutritional Assessment (MNA) and its use in grading the nutritional state of elderly patients. Nutrition.

[65] Zuniga KE, Mackenzie MJ, Kramer A, McAuley E (2016). Subjective memory impairment and well-being in community-dwelling older adults. Psychogeriatrics.

[66] Activities of daily living (ADLs). PhenX Toolkit: Protocols.

[67] Soysal P, Veronese N, Arik F, Kalan U, Smith L, Isik AT (2019). Mini Nutritional Assessment Scale-Short Form can be useful for frailty screening in older adults. Clin Interv Aging.

[68] Bellg AJ, Borrelli B, Resnick B (2004). Enhancing treatment fidelity in health behavior change studies: best practices and recommendations from the NIH Behavior Change Consortium. Health Psychol.

[69] Killip S, Mahfoud Z, Pearce K (2004). What is an intracluster correlation coefficient? Crucial concepts for primary care researchers. Ann Fam Med.

[70] Harris PA, Taylor R, Thielke R, Payne J, Gonzalez N, Conde JG (2009). Research electronic data capture (REDCap)--a metadata-driven methodology and workflow process for providing translational research informatics support. J Biomed Inform.

[71] Harris PA, Taylor R, Minor BL (2019). The REDCap consortium: Building an international community of software partners. J Biomed Inform.

[72] Amarantos E, Martinez A, Dwyer J (2001). Nutrition and quality of life in older adults. J Gerontol A Biol Sci Med Sci.

[73] Eggersdorfer M, Akobundu U, Bailey RL (2018). Hidden hunger: solutions for America’s aging populations. Nutrients.

[74] Gashi S, Kaspar H, Holtforth MG (2023). Personal benefits of older adults engaging in a participatory action research (PAR) project. J Aging Stud.

[75] (2019). Status of poverty in San Antonio.

